# The Solutions to the Uncertainty Problem of Urban Fractal Dimension Calculation

**DOI:** 10.3390/e21050453

**Published:** 2019-04-30

**Authors:** Yanguang Chen

**Affiliations:** Department of Geography, College of Urban and Environmental Sciences, Peking University, Beijing 100871, China; chenyg@pku.edu.cn

**Keywords:** fractal, prefractal, multifractals, self-affine fractals, fractal cities, fractal dimension measurement

## Abstract

Fractal geometry provides a powerful tool for scale-free spatial analysis of cities, but the fractal dimension calculation results always depend on methods and scopes of the study area. This phenomenon has been puzzling many researchers. This paper is devoted to discussing the problem of uncertainty of fractal dimension estimation and the potential solutions to it. Using regular fractals as archetypes, we can reveal the causes and effects of the diversity of fractal dimension estimation results by analogy. The main factors influencing fractal dimension values of cities include prefractal structure, multi-scaling fractal patterns, and self-affine fractal growth. The solution to the problem is to substitute the real fractal dimension values with comparable fractal dimensions. The main measures are as follows. First, select a proper method for a special fractal study. Second, define a proper study area for a city according to a study aim, or define comparable study areas for different cities. These suggestions may be helpful for the students who take interest in or have already participated in the studies of fractal cities.

## 1. Introduction

A scientific research is involved with two processes: description and understanding. A study often proceeds first by describing how a system works and then by understanding why [1]. Scientific description relies heavily on measurement and mathematical modeling, and scientific explanation is mainly to use observation, experience, and experiment [2]. Mathematical reasoning and systematic controlled experiment represent two bases of great achievements in the development of Western science [3]. The basic method of description is measurement, which forms a link between mathematical modeling and empirical studies [4]. The precondition of effective description by measurement and mathematical modeling is to find a characteristic scale, which always takes on a 1-dimension measure and is termed characteristic length [5,6,7,8,9]. However, for complex systems such as cities or systems of cities, it is difficult or even impossible to find a characteristic scale to make mathematical models or quantitative analyses. In this case, it is an advisable selection to replace characteristic scale with scaling notion.

Fractal geometry provides a powerful tool of scaling analysis for complex systems, and it is useful in both theoretical and empirical researches on cities. A number of studies on fractal cities have verified the effect and power of fractal methods and fractal parameters [10,11,12,13,14,15,16,17,18,19,20,21,22,23,24,25]. Unfortunately, new problems have arisen in recent years, that is, the results of fractal dimension estimation for urban form depend on scope of study area and methods of fractal dimension measurement and calculation [11,12,13,14,15,16,26]. This puzzles many scholars who take interest in fractal cities for a long time. What is the root of this kind of problems? Opinions differ in the academic circle, and different scholars have different viewpoints. This paper is devoted to revealing the possible reasons for the diversity of fractal dimension calculation results in the fractal studies on cities. As a preparation, six concepts are explained in advance as follows: (1) embedding space: the Euclidean space in which a fractal exists; (2) scaling range: the middle straight line segments in log–log plots reflect the relationships between the linear scales of measurement and the corresponding measures, and the slopes of the segments indicate fractal dimension; (3) real fractals: scaling range is infinite, and Lebesgue measure is zero; (4) prefractals: scaling range is finite, and Lebesgue measure is greater than zero; (5) monofractals, or unifractals: there is one scaling process; (6) multifractals: there is more than one scaling process. A viewpoint is that monofractals are mostly concerned with spaces, while multifractals deal with measures [27]. In fact, both monofractals and multifractals can be unified into the same mathematical framework by the ideas from entropy conservation [28]. The remaining parts of this paper are organized as follows. In Section 2, the relationships between measurements and fractal dimension are explained. In Section 3, various methods of fractal dimension estimation are collected and sorted. In Section 4, several related questions are discussed. Finally, the conclusions are drawn by summarizing the main points of this work.

## 2. Measurement and Dimension

### 2.1. Euclidean Measurement and Fractal Dimension

As indicated above, scientific research begins with a description. In order to describe a thing, we should measure its number (for a point set), length (for a line), area (for a surface), or volume (for a body). Using number, length, area, or volume, we can define a measurement such as density and shape index [29]. In this way, the characters of a system can be condensed into a simple number. As Lord Kelvin once pointed out: “When you can measure what you are speaking about, and express it in numbers, you know something about it; but when you cannot measure it, when you cannot express it in numbers, your knowledge is of a meager and unsatisfactory kind.” (Cited from [4], p. 37) Therefore, Edwards Deming said, “In God we trust, all others bring data” (Cited from [30]). Once a number is obtained for a thing by measurement, some uncertainty is eliminated. Then we will say we gain information from it. Information indicates an increase of understanding and resolution of uncertainty [31]. For example, if we want to know the size of a lake, we can measure its area by means of an electronic map. The smaller the scale of measurement, the more accurate the result of measurement. If the scale becomes smaller and smaller, and the measurement results converges rapidly to a certain value, then we can say that the lake area has a characteristic scale. The characteristic length can be represented by the radius of the lake’s equivalent circle [4].

In urban geography, urban land use area can be used to reflect the extent of space filling. However, if we try to measure the total area of land use of a city, the process will become very complicated. First, it is hard to identify a clear urban boundary line. Second, the patches of urban land use on remoting sense images are too random, fragmented, and irregular to be exactly caught. Although the scale of measurement becomes smaller and smaller, the measurement results will never converge. Finally, the measurement process reaches the limit allowed by image resolution and is cut off. The essence of the problem from image resolution can be treated as a type of the finite size effect in fractal measurements. So, in order to characterize the land-use filling degree of urban space, we must find a new approach. According to fractal theory, urban area can be replaced by a scaling exponent, that is, fractal dimension. By the double logarithmic linear relation between scales (say, linear sizes of boxes) and corresponding measures (say, numbers of nonempty boxes), we can estimate a slope on a log–log plot [16,32]. The value of the slope indicates the fractal dimension, and the parameter can reflect the space filling extent of urban land use.

It can be seen that there is a symmetry and duality relation between Euclidean geometry and fractal geometry. It is just Euclidean geometric measurement that leads to fractal dimensions. For a Euclidean geometry body, the dimension is known: 0 dimension for points, 1 dimension for lines, 2 dimensions for surfaces, and 3 dimensions for bodies. Generally speaking, without measurement, we cannot know the length, area, or volume. In contrast, for a fractal object defined in an embedding space with given Euclidean dimension *d*_E_, the length, or area, or volume, is known in principle: if the topological dimension *d*_T_ is 1, the length is infinite; if the topological dimension *d*_T_ is 0, the length, or area, or volume, is 0. In theory, the Lebesgue measure of a real fractal object is zero. This suggests that a fractal defined in a 2-dimensional embedding space (*d*_E_ = 2) have no area, and a fractal defined in a 3-dimensional embedding space (*d*_E_ = 3) has no volume. However, without measurement and calculation, we cannot know its fractal dimension value. In the process of fractal measurement, common scales are always replaced by scaling, and the conventional measures such as length, area, and volume are replaced by fractal dimension. Where urban form is concerned, both area and fractal dimension can reflect space filling extent. If the area measurement of a city fails, we will use fractal dimension to replace the area to characterize it space filling.

### 2.2. Uniqueness and Diversity of Fractal Dimension

A geometric phenomenon has only one value of dimension, which is of Euclidean dimension or fractal dimension. In other words, for a given aspect of a geometric object, the dimension value is uniquely determined. For example, for a circle or a square, it has two aspects: area and perimeter. The dimension for area is 2, and the dimension for boundary line is 1. Where a fractal is concerned, thing seems to be complicated, but can be made clear. For a regular fractal defined in 1-dimension embedding space and its topological dimension is 0 (e.g., Cantor set), or for a fractal line defined in 2-dimension embedding space but its topological dimension equals 1 (e.g., Koch curve, Peano curve), it has only one aspect and the fractal dimension is uniquely determined in theory. For a fractal object defined in 2-dimension embedding space, which bears two topological dimensions *d*_T_: 0 for point sets and 1 for boundary lines (e.g., Box growing fractal, Sierpinski gasket), it has two aspects corresponding to two different fractal dimensions. As a special example, Vicsek fractal (box growing fractal) has fractal area (aspect 1, *d*_T_ = 0) and fractal boundary (aspect 2, *d*_T_ = 1), both the two fractal dimension values are *D* = ln(5)/ln(3) = 1.465 [33]; Sierpinski gasket also has two aspects, the fractal dimension of fractal area (aspect 1, *d*_T_ = 0) is *D* = ln(3)/ln(2) = 1.585 [5], while the fractal dimension of fractal boundary (aspect 2, *d*_T_ = 1) is *D* = ln(5)/ln(2) = 2.3219 [34]. Please note that the similarity dimension and radial dimension can exceed the dimension value of its embedding space; in contrast, the box dimension must come between the topological dimension *d*_T_ and the Euclidean dimension of the embedding space *d*_E_ [12,14,16,28,32]. The Koch lake is a special case, which also has two aspects: area and perimeter. The dimension for area is 2, and the dimension for boundary curve is 1.2619 [5]. The perimeter of the Koch lake is a fractal line (*d*_T_ = 1), but the area within the perimeter is a Euclidean plane (*d*_T_ = 2).

Fractal studies on cities are neither pure mathematical processes nor absolute true portrayal of cities. Just like any other scientific research, urban studies based on fractal geometry are involved with three worlds, that is, real world, mathematical world, and computational world [35]. The mathematical world (objective world) is always linked to the real world (objective world) by the computational world (subjective world). Random fractals in the real world are more complex than the regular fractals in the mathematical world. For a fractal city defined in the 2-dimension space, if we examine the land-use pattern, it will be involved with two aspects, urban area and urban boundary. The fractal dimension of urban form can be estimated by the box-counting method [12,14,16,36,37,38], sandbox method [10], area–radius scaling (cluster growing) method [11,39,40,41,42], density–radius scaling method [43], length–area scaling [44], correlation and dilation methods [45], wave-spectrum scaling method [46], and so on; the fractal dimension of boundary line can be directly estimated by walking-divider method [47], or indirectly estimated by perimeter–area scaling method [48,49,50], and so on. Unfortunately, in empirical studies on city fractals, the process is very complicated. Differing from the field investigation in the real world and logic reasoning in the mathematical world, the fractal dimension estimation is conducted in the computational world. However, the subjectivity of fractal dimension measurement and calculation is not the fatal problem; the key lies in the fractal properties of cities, and this will be discussed in next section.

### 2.3. Varied Fractal Dimension Calculation Methods

In order to calculate fractal dimension, fractal scientists propose a number of methods. The most of these methods are generic in different fields, and they were summarized and sorted by fractal experts years ago [8]. In urban studies, fractal dimension estimation methods were once researched, developed, and sorted by urban theoreticians such as Michael Batty, Frankhauser, and Paul Longley [11,39]. Generally speaking, different methods have different uses, but sometimes, we can use different methods to estimate the same fractal dimension. For example, at least four types of methods can be used to estimate the fractal dimension geographical boundaries [11,50]. Now, various fractal dimension methods have been introduced into fractal city studies.

First, different methods can be employed to compute the fractal dimensions for different aspects of a city or a system of cities. Based on time series of urban evolution, the fractal dimensions of dynamic processes can be estimated; based on spatial datasets, the fractal dimensions of spatial structure of cities can be estimated; based on sectional data of cities, the fractal dimensions of rank-size distributions and hierarchies can be estimated (Table 1). The datasets of time series, space series, and hierarchy series can be used to make different types of spatial analyses and correlation analyses for urban studies [51].

Second, different methods can be utilized to calculate different types of urban fractal dimension. In the majority of cases, we compute the self-similar fractal dimensions of urban form or urban systems, but sometimes, we are concerned about the self-affine fractal dimension of urban growth [52]. Self-similar fractal dimension and self-affine fractal dimension represent two different but related fractal parameters (Table 2). Self-similar growth is isotropic growth, while self-affine growth indicates anisotropic growth [33]. Generally speaking, the density distribution of urban transport network takes on self-similar growth with isotropy, while urban population and land-use expansion takes on self-affine growth with anisotropy.

Third, a variety of methods can be used to compute the same fractal dimension of cities. The solution to a problem is not the only one. For each aspect of a city fractal, more than one approaches can be used to estimate the fractal dimension (Table 3). For example, we can use the walking-divider method, perimeter–area scaling method, and box-counting method to estimate the fractal dimension of urban boundary [11,47,50,53]. In theory, the three methods are equivalent to each other. An aspect of a fractal object has only one fractal dimension. However, in practice, the calculation results from different methods are not always consistent with each other. This gives rise to a number of problems and different opinions about fractal dimension calculation of cities.

## 3. Solutions to Fractal Dimension Estimation Problems

### 3.1. A Dilemma of Fractal Dimension Estimation

To make or use a mathematical model, we must find an effective algorithm and approach to determine its parameter values. The algorithms include the ordinary least squares (OLS), maximum likelihood estimation (MLE), and major axis method (MAM). A number of measurement approaches, as displayed above, are proposed in literature to estimate fractal dimension values (Table 1, Table 2 and Table 3). Generally speaking, different methods are applied to different directions (different aspects or properties). For example, the walking-divider method can be used to estimate the fractal dimension of urban boundary dimension rather than urban area; power spectrum is used to research the urban evolution based on time series rather than urban form based on spatial data; fractional Brownian motion (FBM) is used to estimate self-affine record dimension rather than self-similar trail dimension; the sandbox method, clustering growing, and wave-spectrum are used to calculate the radial dimension for characterizing urban growth; the box-counting method is used to compute fractal dimensions for describing spatial structure and texture of urban morphology, and so on. Sometimes, several different methods can be applied to the same aspect of cities. For example, the box-counting method, area–radius scaling method, sandbox method, and wave spectrum analysis based on density–radius scaling can be employed to estimate the fractal dimension of urbanized area. In theory, a fractal aspect has only one fractal dimension value, but unfortunately, in empirical studies, different methods often result in different fractal dimension estimation values, and in many cases, the numerical differences are statistically significant and cannot be ignored in a spatial analysis. Even for a given method, a fractal dimension value often depends on the size and central location of the study area defined by a researcher. This is involved with the uncertainty of fractal dimension calculation, which puzzles many fractal scientists.

A simple prototype is helpful for understanding complex phenomena in scientific research. In order to study the atomic structure, physicists first explored the structure of the simplest atom, the hydrogen atom; in order to study the structure of viruses, biologists first concentrated on exploring the structure of simple virus, bacteriophages. Simple prototypes often form the beginning of theoretical analysis. To reveal the root of the problem of uncertainty in fractal dimension calculation, we can examine two regular fractals, including monofractal and multifractal patterns. All these regular fractals reflect prefractal structure because we can never look the real fractal patterns. The real fractals in geometry are just like the high-dimensional spaces in linear algebra, which can be imagined but can never be observed. All of the fractal images we encounter in books or articles represent prefractals rather than real fractals [54]. The difference between real fractals and prefractals is as follows: A real fractal has infinite levels, but a prefractal is a limited hierarchy; therefore, the Lebesgue measure of a real fractal equals 0, but the Lebesgue measure of a prefractal is not equal to 0. For a given aspect (say, area or boundary) of a regular monofractal object, we can apply different methods to its prefractal structure to determine its fractal dimension. Different methods lead to the same result, which represents the real fractal dimension value. However, for a multifractal object, the real fractal dimension cannot be computed by applying some method to its prefractal pattern. We can only obtain comparable parameters rather than real fractal dimension for multifractal systems.

**Figure 1:**
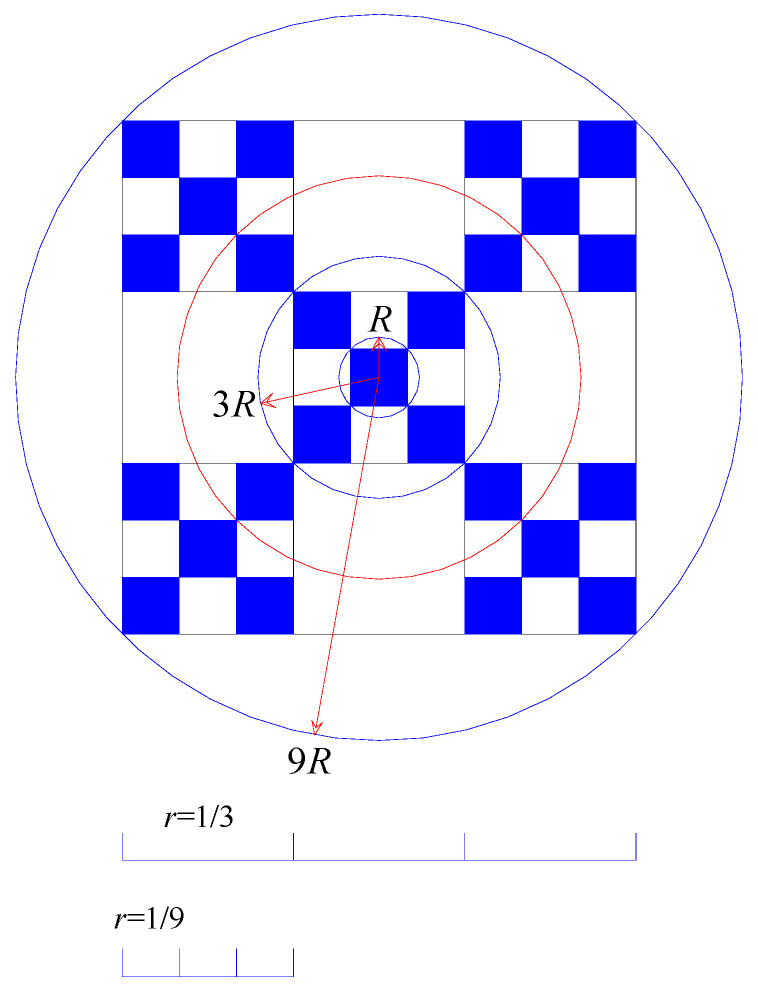
Three approaches to estimating fractal dimension of a regular fractal (the first 3 steps). Note: The schematic diagram of measurement method is drawn by referring to the work of Batty and Longley [11]. Sandbox method, radius–number scaling, and box-counting method can be employed to calculate the fractal dimension of this growing fractal.

By analyzing the regular fractal objects, we can gain new insight into fractal structure and fractal dimension measurement. First of all, let us see a simple regular growing fractal, which is employed to model urban growth in literature [11,21,39,42,55]. This fractal was proposed by Jullien and Botet [56] and became well known due to the work of Vicsek [33], and it is also termed Vicsek’s figure or box fractal (Figure 1). Three approaches can be applied to its prefractal pattern, including the box-counting method, sandbox method, and cluster growing scaling method. The third approached can be divided into two equivalent methods: area (number)–radius scaling and density–radius scaling. According to its regular composition, we can obtain the datasets comprising the first 10 steps (Table 4). Based on the box-counting method, sandbox method, and area–radius scaling method, the scaling exponent is just its fractal dimension, and the value is *D* = ln(5)/ln(3) = 1.465. Based on the density–radius scaling method, the scaling exponent is *a* = 2 − *D* = 0.535, and thus the fractal dimension is also *D* = 2 − 0.535 = 1.465. This value is exactly the real fractal dimension of this fractal object.

**Figure 2:**
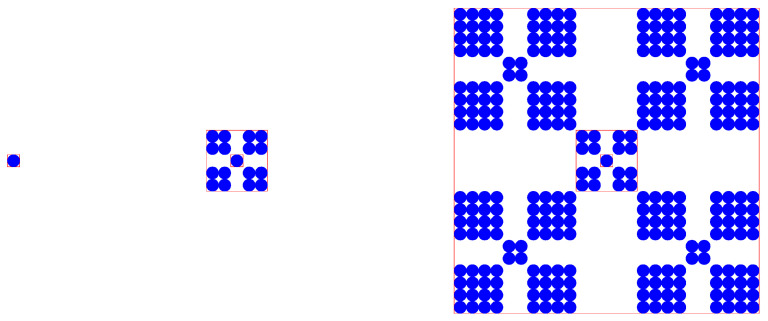
The prefractal structure of a regular growing multifractals (the first three steps). Note: The fractal pattern is adapted by referring to the work of Vicsek [33], but the figure is designed by the author. This fractal can be used to model multifractal growth of cities [14,57].

Further, let us examine a regular growing multifractal object, which reflects the pattern of spatial heterogeneity. This fractal is presented by Vicsek [33]. It can be used to model multifractal growth of cities [57]. The first three steps represent a prefractal process (Figure 2). The box-counting method can be used to calculate its global dimension. Step 1: fractal dimension *D* = 0 (for a point, the fractal dimension can be obtained by L’Hospital’s rule). Step 2: box dimension *D* = −ln(17)/ln(1/5) = 1.7604. Step 3: box dimension *D* = −ln(289)/ln(1/25) = 1.7604. If we apply the sandbox method to the figure in the third step, the fractal dimension is also *D* = 1.7604. However, two problems can be found by careful investigation. First, different fractal units bear different fractal dimension values. One of basic properties of fractals, including monofractals and multifractals, is entropy conservation: different fractal units at a given level has the same Shannon entropy value [9,28,58,59,60,61]. In fact, different fractals, except fat fractals, can be unified into the same framework [28], and expressed by a transcendental equation as below [60,61]

(1) $$\sum_{i=1}^{N(r)}P_{i}{\left(r\right)}^{q}r_{i}^{(1-q)D_{q}}=1,$$

in which *P_i_* denotes the growth probability of the *i*th fractal unit, *r_i_* represents the linear size of the *i*th fractal unit, *q* refers to the order of moment, and the power exponent *D_q_* is termed the generalized correlation dimension [59]. For monofractals, we have, *D_q_* ≡ *D*_0_; for self-affine fractals, different directions have different fractal dimension values, and for a given direction, we have *D_q_* = *D*_0_; for multifractals, different parts of a multifractal system have different local fractal dimension values, and the global fractal dimension *D_q_* depends on the moment order *q* [28]. Equation (1) can be employed to identify different fractals from varied complex systems. One of the commonalities of different fractals is the conservation of entropy, which can be derived from Equation (1). However, the fractal dimension does not comply with a conservation law. In fact, for a multifractal system, different parts have different local fractal dimensions. For example, for the second level of the third step, the five parts have two fractal dimension values. The central part, box dimension is *D* = ln(1/17)/ln(1/5) = 1.7604; the other four parts, box dimension is *D* = ln(4/17)/ln(2/5) = 1.5791. Second, the parameter value estimated by the box-counting method and the sandbox method is not equal to its real dimension value. In theory, the calculated values represent the capacity dimension of this multifractals, i.e., *D*_0_ = 1.7604. The regular multifractal structure can be modeled by a transcendental equation based on probabilities and the corresponding scales. Where the third step is concerned, the multifractal transcendental equation can be constructed as follows

(2) $$\sum_{i=1}^{5}P_{i}^{q}r_{i}^{(1-q)D_{q}}={\left(\frac{1}{17}\right)}^{q}{\left(\frac{1}{5}\right)}^{(1-q)D_{q}}+4{\left(\frac{4}{17}\right)}^{q}{\left(\frac{2}{5}\right)}^{(1-q)D_{q}}=1,$$

Using Matlab to find its numerical solutions, we can obtain its multifractal parameter values (Table 5). The results show that the real capacity dimension is about *D* = 1.5995 < *D* = 1.7604. The capacity dimension based on the box-counting method and the sandbox method is in fact the maximum dimension, that is *D*_−∞_ = 1.7604. The capacity (*D*_0_) is the maximum value of the local dimension, while the maximum dimension (*D*_−∞_) is the upper range value of global dimension.

Now, a basic judgment can be reached as follows. For a regular monofractal (Figure 1), the real fractal dimension value can be calculated by the prefractal structure. However, for a regular multifractal (Figure 2), the real fractal dimension values cannot be obtained by applying some method such as the box-counting method to its prefractal structure. This suggests that the resolution of remote sensing images influences the multifractal parameter estimation of cities, reminding us of the finite size effect in fractal measurements. In fact, for a random multifractal system, we cannot construct its multi-scaling transcendental equation such as in Equation (1). As a result, we will never know the real fractal parameter values. We can only estimate a set of comparable parameter values to replace the real values. In the real world, fractal cities have two properties. First, they are random multifractals rather the regular monofractals or regular multifractals; second, they only develop prefractal structure rather than real fractal structure. What is more, the prefractal structure of multifractals are always mixed up with self-affine processes, fat fractal components, or even fractal complements. In this case, the processes of measurements and analyses become very complicated relative to the regular fractals.

### 3.2. The Reasons for the Divergence of Calculation Results

In urban studies, the fractal dimension value is always influenced by the selection of method and the definition of the study area. Fractal dimension estimation depends on the method, and this is indeed a problem. However, study area (size, location) influences fractal dimension values, but this seems not to be a problem. The concrete reasons are as follows (Table 6). (1) Prefractal is the main reason for the influence of method on the fractal dimension values. For a real fractal, its scaling range is infinite; for a prefractal, its scaling range is limited to certain scales. The precondition of accurate calculation of fractal dimension for a random fractal is that the scale of measurement is close to infinitesimal in theory. For a given aspect of a given random fractal, if the measurement scale becomes smaller and smaller, different methods will lead to the similar fractal dimension values. However, for a prefractal, the linear size of measurement scale is limited to its lower bound of scaling range and cannot approach infinitely small scales. (2) Multifractal structure is the main reason for the influence of the size and central location of the study area on the fractal dimension values. The fractals in the real world are all random multifractals rather than monofractals. A monofractal object has only one scaling process, while multifractals have more than one scaling process. For a monofractal object, capacity dimension is equal to information dimension and correlation dimension, and global dimension is equal to local dimension. However, for a random multifractal, the capacity dimension is often greater than the information dimension, and the information dimension is often greater than the correlation dimension, and so on [28,33,59]. Different parts of a random multifractal object have different local fractal dimension, and different sizes of study area yield different fractal parameters. Thus, if we define different study area for a multifractal city, the results of fractal dimension estimation will be different. (3) Self-affine fractal process is a reason associated with the influence of both method and study area on fractal dimension estimation. Self-similar growth indicates isotropy, and measurement direction does not influence fractal dimension estimation, while self-affine growth implies anisotropy, and different measurement directions lead to different fractal dimension. Especially, self-affine growth causes area–radius scaling to break, and form what is called bi-fractal pattern in a log–log plot. The essence of bi-fractals rests with self-affine development and growth of fractal systems, which can be illustrated by testing the regular self-affine fractals.

### 3.3. Solutions to Problems

In the literature, the word “solution” has two basic meanings: one is finding a way of solving a problem or dealing with a difficult situation, and the other is an answer to a puzzle or to a problem in mathematics. In this paper, I do not provide an answer to a puzzle/problem of fractal dimension calculation; instead, I discuss the ways of solving the uncertainty problem of fractal dimension measurements. The problems cannot be solved once and for all. Different problems should be treated differently, and different types of problems need different types of solutions (Table 7). There are two main ideas to solve the problem: one is to find a proper method for a special studies on fractal cities, and the other is to replace the real fractal parameters with the comparable fractal parameters. Based on the above explanation, the possible solutions to the problems of fractal dimension estimation of cities can be presented as follows.

First, the solutions to the problem of method dependence of fractal dimension estimation. The results of model parameter estimation depend on the methods, and this phenomenon is not only in the field of fractal research. It is hard to find good solutions to this kind of problem. In fact, for a random system or based on random variables, it is unlikely to find the true parameter values for its mathematical models. Scientists then look for comparable parameter values instead of real parameter values (Faute de mieux). In urban studies on fractals, two approaches are employed to deal with this kind of problems. One approach is to replace the real fractal values by comparable fractal dimension values which are based on the same criterion for different times, spaces, levels, and scales; the other approach is to find the most suitable method for specific research objectives. As indicated above, different methods have different merits and can be applied to different aspects and directions of urban studies (Table 1, Table 2 and Table 3). If we measure the fractal dimension of geographical fractal lines such as urban boundaries, the advisable methods are the walking-divider method and the area–perimeter scaling method [11,47,50]; if we research complex patterns and spatial structure, we can utilize the box-counting method [12,14,16,37]; if we explore the dynamic process of isotropic urban growth, we should adopt the sandbox method, area–radius scaling, and density–radius scaling [10,11]; if we examine anisotropic urban growth, we should adopt wave-spectrum analysis based on density–radius scaling [34,46]. Generally speaking, in order to estimate the fractal dimension of an urban boundary, we often regard urban area as Euclidean surface with an integral dimension *d* = 2 [11]. Thus, we can make use of the area–perimeter scaling method [48,59]. However, if we try to compare the fractal dimension value of urban form with that of urban boundary for the same city, we should adopt the box-counting method, which can give comparable fractal dimension for both boundary line and urban pattern within this boundary.

Second, the solutions to the problems of study are scope dependence of fractal dimension calculation. A random prefractal object has a limited scaling range, in which fractal property appears. As shown above, for a given aspect (area or boundary) of a regular monofractal, its fractal dimension value is unique, and the real fractal dimension can be calculated through its prefractal structure. However, for a regular multifractal object, different parts have different local fractal dimension values, and the real fractal dimension values cannot be computed by its prefractal structure. No regular monofractal can be found in the real world. A real city is a random multifractal system with prefractal structure. It is impossible to identify the boundaries of different fractal units. Different sizes of study area process different global fractal dimensions, and different parts have different local fractal dimensions. Consequently, based on different scope (size and central location) of a study area, different fractal parameter values will be worked out. We never know the sets of real fractal dimension values. In this case, we can use relative comparable parameter values instead of absolute real parameter values. In particular, we can employ multifractal dimension spectrums to make spatial analyses for cities and systems of cities. To obtain comparable fractal dimension, we must define a comparable study area. Where urban form and box-counting method are concerned, the procedure is as follows: (1) use a proper method and the concept from characteristic scales to define objective urban boundaries; (2) define a measure area based on certain direction for the urban envelope; (3) use the measure area as the maximum box for box-counting. In the specific research, the methods should be adjusted according to specific problems and research objectives.

## 4. Questions and Discussion

In scientific research, if we cannot obtain absolute measurements based on certain values, we should try to find the relative measurements based on comparable values. If we only focus on the fractal studies on cities, the uncertainty of fractal dimension estimation is a problem; however, if we look at the entire system of scientific methodology, this kind of uncertainty is not a problem. In fact, the uncertainty of model parameter estimation is a common phenomenon in scientific research. Mathematical models and quantitative analyses can be divided into two types: one is based on characteristic scales, and the other is based on scaling. The traditional mathematical tools are mainly based on characteristic scales, while fractal studies are mainly based on scaling. In conventional mathematical modeling processes, the parameter estimation relies heavily on computational methods. It is impossible to evaluate the real parameters for the great majority of mathematical models by empirical analysis. A number of examples are listed as follows (Table 8). (1) For the simplest linear regression model, a number of algorithms such as the least squares method, maximum likelihood method, major axis method, and reduced major axis method can be employed to estimate the regression coefficients, and different methods lead to different results. Moreover, sample size and variable dimension also influence the constant and regression coefficients. (2) For factor analysis, the calculation results depend on the methods of factor extraction and factor rotation, and there are various methods for factor extraction (e.g., orthogonal transformation, maximum likelihood) and rotation (e.g., Quartimax, Varimax). What is more, the starting point of factor analysis can be correlation coefficient matrix or covariance matrix, and different starting points lead to different numerical results. (3) For hierarchical cluster, a final output depends on the methods of cluster and measure, and there are various methods for cluster (e.g., between-groups linkage, within-groups linkage) and measure (e.g., Euclidean distance, Pearson correlation). Different measure methods lead to different proximity matrixes, and different cluster methods based on different proximity matrixes lead to different final results. Moreover, the value transform methods (e.g., standardization, normalization, etc.) influence cluster analysis. (4) For auto-regression analysis based on time series, there are various methods for parameter estimation; for spatial autocorrelation analysis, different impedance functions lead to different contiguity matrixes, which in turn lead to different Moran’s *I*, Geary’s *C*, Getis’s *G*, and so on.

The uncertainty of fractal dimension calculation is associated with the uncertainty of scaling exponent estimation. Scaling is one of basic properties of fractals. If and only if a scaling phenomenon satisfies three conditions, it can be regarded as a fractal set. The conditions include scaling law (scale invariance), fractal dimension (Hausdorff dimension is greater than its topological dimension), and entropy conservation (the Shannon entropy of each fractal units is a constant) [28]. However, many natural and social complex systems follow scaling law, but have no fractal dimension and do not meet the entropy conservation condition. These types of complex systems cannot be effectively modeled by traditional mathematical methods. In this case, we can use scaling exponents to characterize the complex systems. In recent years, scaling has become a hot topic in urban studies, and a number of interesting research results emerged [62,63,64,65,66,67,68]. Among various urban scaling, the most frequently appearance is the allometric scaling. However, in empirical studies, it is difficult to obtain stable scaling exponent values. Algorithms, study area, datasets, scaling ranges, and so on, influence the results of scaling exponent estimation [69]. A recent discovery is that the scaling exponent values of the allometric relation between patents and city sizes depend on the population size cut-offs [70]; Another meaningful discovery is that the scaling exponent values of the allometric relation between urban CO_2_ emissions and city population sizes depend on the definition of urban area [71,72]. A scaling exponent is often directly or indirectly related to fractal dimension. The allometric scaling exponent is actually the ratio of one fractal dimension to another fractal dimension [69]. In this sense, the uncertainty of fractal dimension computation account for the uncertainty of scaling exponent estimation of cities.

As indicated above, fractal dimension calculation is implemented in the computational world rather than in the mathematical world. Cities appearing in the real world are objective, but it is hard to reveal the deep structure and the complicated relationships between causes and effects hidden behind urban world. Regular fractals, fractal laws, and strict logic reasoning defined in the mathematical world are also objective, but the graceful mathematical processes are not consistent with the real systems. We can use Koch snowflake to model the central place system of human settlements, but the real central place networks differ from the ideal Koch snowflake pattern. We can employ the diffusion-limited aggregation (DLA) models to simulate urban growth and form, but real urban evolution differs from the DLA process. So, Albert Einstein once said, “I don’t believe in mathematics.” He observed, “As far as the laws of mathematics refer to reality, they are not certain, and as far as they are certain, they do not refer to reality.” In fact, as a pure theoretical physicist, Einstein ignored an important linkage, which represents a logic bridge between mathematical world (e.g., theoretical models and laws) and real world (e.g., urban growth and form). The bridge coming between reality and mathematics is what is called computational world, which is a subjective world to some extent. Spatial measurements, data processing, algorithms, and so on, are all defined in the computational world. The ways of measurements and data extraction as well as choices of algorithms and models varies from person to person. Therefore, for the urban form of a same city, the fractal dimension estimation results may be different from one another significantly. The more experienced a scholar is, the better the process of fractal dimension calculation is handled. However, no matter how hard we try, we can never obtain the real values or absolutely exact values for the fractal parameters of a city. The best results that we can gain in a study are a set of comparable fractal dimension values for different times, places, levels, or scales.

The three worlds are related to three types of studies about fractal cities. According to the theory of systems analysis, academic research falls into three categories: behavioral research, normative research, and values research [73]. Behavioral research on fractal cities are a type of positive studies, which correspond to the real world; normative research on fractal cities are pure theoretical studies, which correspond to the mathematical world. On the one hand, fractal geometry is a powerful tool for exploring nonlinear processes, irregular patterns, and scale-free distributions (spatial distributions and probability distributions), and can be used to bright to light the evolution process and spatial pattern of cities. On the other, a fractal suggests an unlimited filling process in a limited space, which leads to an optimized pattern. In this sense, fractals represent the optimum designs in nature and society. A fractal object can occupy its space in the most efficient way. Fractal geometry can be devoted to finding the most reasonable structure of urban systems or construct ideal models for urban spatial analyses. The two types of research, behavioral research and normative research, can be linked by the values research. For fractal cities, the values research is to develop a set of evaluation indexes, by which we can judge the pros and cons of the development of a city in the past and at present (for behavioral research) and its direction of optimal design in the future (for normative research).

The emergence of fractal geometry represents a discovery of new form of symmetry, i.e., scaling symmetry. The basic property of a fractal is its invariance under contraction or dilation [74]. Because of the scaling symmetry, it is impossible to find certain length, area, volume, and number for a scale-free system. In this case, we can use a scaling exponent to replace the common measures. One of the basic scaling exponents is fractal dimension. As a matter of fact, there must be some symmetry when there is immeasurable quantity [75]. The fractal concept came from the immeasurable length of the cost of Britain [76]. Today, fractal dimension seems to be another immeasurable quantity. Although we found various factors that affect fractal dimension measurements, there is no exclusion of the possibility that a kind of super symmetry is hidden behind the scaling symmetry [13]. What is more, spatial autocorrelation of urban patterns influences spatial measurement results. Reliable measurements depend on no spatial autocorrelation. These problems remain to be explored in future studies. All in all, we cannot give up eating for fear of choking, and cannot give up fractal geometry in urban studies because of the uncertainty of fractal dimension estimation. The application value of a measure or parameter value lies in comparability rather than reality or accuracy. It is like a small-scale map of a country or the world. A map is a typical model, and the mapping is a typical process of model building [77]. When we map the geographical things on the three-dimensional spherical surface to the two-dimensional plane, in any case, we will encounter the projection deformation, which results in the distortion of the spatial pattern on the map. However, the maps are very useful in everyday life and geographical research.

## 5. Conclusions

There are various approaches to fractal dimension estimation, and the great majority of them can be adopted to research fractal cities. Generally speaking, different methods are suitable for different directions of urban studies. Sometimes, several different methods can be applied to the same aspect of fractal dimension estimation, but the results are different from each other significantly. What is more, changing the scope of study area for a city, the result will change accordingly. This gives rise to a dilemma of fractal dimension calculation, that is, fractal dimension values depend on both methods adopted and scope of study area defined in an empirical analysis. The main factors influencing fractal dimension calculation include prefractal structure, multifractal patterns, and self-affine fractal growth. The concrete reasons can be summarized as follows. First, random prefractal structure result in diversity of fractal dimension estimation based on different methods and the deviation of estimated fractal dimension values from real fractal dimension values. The regular monofractal dimension can be determined by its prefractal, but the dimension of a random fractal cannot be evaluated by its prefractal structure. The precondition of calculating its real or exact fractal dimension values lies in the linear scales of spatial measurement approaching to infinitely small size. If the linear size of spatial measurement is small enough, different methods will lead to the similar or even the same fractal dimension values. Unfortunately, due to the limited scaling ranges of random prefractals, the linear size of spatial measurement is confined to a certain range. Second, random multifractal patterns result in the deviation of estimated fractal dimension values from the real fractal dimension values and the dependence of fractal dimension values on the scope of study area. On the one hand, the global fractal dimension of a (regular or random) multifractal system cannot be determined by its prefractal structure. However, in practice, we can only face the prefractal structure rather than real fractal structure of random multi-scaling fractals. On the other hand, multifractals bear spatial heterogeneity and different parts have different local fractal dimension values. Consequently, changing the size or the central location of study area results in different fractal dimension calculation results. Third, self-affine fractal growth influences the fractal dimension estimation. A self-similar growing fractal bears isotropic pattern and its fractal dimension can be estimated by area–radius scaling or density–radius scaling. However, many fractal growing processes of cities take on self-affinity and anisotropy. In this case, the scaling range often break into two segments, and it is difficult to find the reliable fractal dimension values. Because of the interaction between random patterns, prefractal structure, multi-scaling processes, and self-affine growth, things become very complicated and the fractal dimension values take on diversity in an urban study. In addition, the spatial autocorrelation of geographical phenomena is one of the possible influencing factors of fractal dimension measurement results. However, this problem is more of an algorithmic problem than the topic discussed in this article. Therefore, there is no in-depth discussion on this issue for the time being. The dependence of fractal dimension values on scope of study area is indeed a problem, but strictly speaking, the dependence of the fractal dimension on methods is not a problem. In mathematical modeling and quantitative analysis, the method-dependence of model parameter values is a common phenomenon. The solution to the problems lies in two aspects. On the one hand, one must find the most proper method for the special aspect of a city fractal and for the special direction of a study; on the other, we can use the comparable fractal dimension values to replace the real or exact fractal dimension values.

## Figures and Tables

**Table 1:** Methods of urban fractal dimension estimation based on time series, spatial structure, and hierarchical structure.

Object	Method	Fractal Dimension
Time series (process)	Power spectrum	Self-affine and self-similar dimension
	Reconstructing phase space	Correlation dimension
	Elasticity relation	Similarity dimension
	……	……
Spatial structure, texture, and distribution (pattern)	Box counting method	Self-similar dimension
	Sandbox	Self-similar dimension
	Radius scaling (cluster growing)	Self-similar dimension
	Wave spectrum	Self-affine and self-similar dimension
	Walking-divider method	Self-similar dimension
	Perimeter–area scaling	Self-similar dimension
	……	……
Hierarchical structure (cascade), Scale-free network	Size distribution	Self-similar dimension
	Hierarchical scaling	Self-similar dimension
	Allometric scaling	Self-similar dimension
	Renormalization	Self-similar dimension
	……	……

**Table 2:** Fractal dimension estimation methods for self-similar patterns and self-affine processes of cities.

Fractality	Aspect	Method
Self-similarity	Area/Point (spatial structure)	Box counting method
		Prism counting method
		Area–radius scaling (cluster growing)
		Sandbox method
		Wave spectrum analysis
		……
	Boundary/Line (spatial texture)	Walking-divider method
		Perimeter–area scaling
		……
	Network	Renormalization
		……
Self-affinity	Area/Line	Fractional Brownian Motion (FBM)
		Wave spectrum
		……
Multifractality	Points/Lines/Areas	Renyi entropy measurement and Legendre transform
		Reconstruction of probability (*μ*-weight method)
		Wavelet analysis
		……

**Table 3:** Direct and indirect fractal dimension estimation methods for cities.

Property	Method (Type)	Method (Subtype)
Direct	Box counting	Common box, prism box, sandbox
	Radius scaling (cluster growing)	Area–radius scaling, number–radius scaling, density–radius scaling, radius of gyration
	Walking-divider	Various step length processes
	……	……
Indirect	Spectral analysis	Wave spectrum, power spectrum
	Geometric measure relation	Allometric scaling, perimeter–area scaling, length–area scaling, elasticity relation
	Fractional Brownian Motion	(mainly for self-affine process)
	……	……

**Table 4:** Box-counting method, sandbox method, and cluster radius scaling methods for fractal dimension of a regular monofractal growing fractal.

Level	Box-Counting Method		Sandbox Method		Cluster Growing and Radius Scaling		
*m*	Box Side Length *r*	Box Number *N*(*r*)	Sandbox Side Length *L*	Box Number *N*(*L*)	Radius *R*	Fractal Unit Number *N*(*R*)	Density *ρ*(*R*)
0	1.0000	1	1	1	0.7071	1	1.0000
1	0.3333	5	3	5	2.1213	5	0.5556
2	0.1111	25	9	25	6.3640	25	0.3086
3	0.0370	125	27	125	19.0919	125	0.1715
4	0.0123	625	81	625	57.2756	625	0.0953
5	0.0041	3125	243	3125	171.8269	3125	0.0529
6	0.0014	15,625	729	15,625	515.4808	15,625	0.0294
7	0.0005	78,125	2187	78,125	1546.4425	78,125	0.0163
8	0.0002	390,625	6561	390,625	4639.3276	390,625	0.0091
9	0.0001	1,953,125	19,683	1,953,125	13,917.9828	1,953,125	0.0050
…	…	…	…	…	…	…	…

**Table 5:** Four sets of fractal parameters of a regular growing multifractal (typical values).

Moment Order *q*	Global Parameters		Local Parameters	
	Generalized Correlation Dimension *D_q_*	Mass Exponent *τ_q_*	Singularity Exponent *α*(*q*)	Local fractal Dimension *f*(*α*(*q*))
−100	1.7429	−176.0374	1.7604	0.0000
−10	1.6404	−18.0440	1.6933	1.1107
−2	1.6054	−4.8161	1.6153	1.5855
−1	1.6022	−3.2044	1.6081	1.5963
0	1.5995	−1.5995	1.6020	1.5995
1	1.5970	0.0000	1.5970	1.5970
2	1.5949	1.5949	1.5930	1.5910
10	1.5859	14.2730	1.5806	1.5330
100	1.5798	156.3975	1.5791	1.5129

Note: Multifractal parameters include global parameters and local parameters. The former comprises generalized correlation dimension and mass exponent, while the latter consists of local fractal dimension and singularity exponent. Global parameters describe the spatial dependence of multifractal elements from a global perspective, while local parameters describe the spatial heterogeneity of multifractal distributions from a local perspective. See [7,13,14,27,28,33,58,59,60].

**Table 6:** Three significant properties of city fractals: prefractal structure, multifractal form, and self-affine growth.

City Fractal	Theoretical Problem	Practical Problem
Random prefractal	The range of measurement is limited. The topological dimension is easily misunderstood, and this leads to misunderstanding on scaling range.	Finite size effect influences the identification of patterns, which in turn influence fractal dimension estimation.
Random multifractal	Different moment order *q* lead to different global fractal dimensions, different parts have different local fractal dimensions.	The scope of study area and the angle of view influence the multifractal parameter spectrums.
Random self-affine fractal	Anisotropic growth lead to different fractal dimension values in different directions.	It is hard to estimate fractal dimension using radius scaling method.

**Table 7:** The possible directions of solving problems in fractal dimension estimation.

Factor		Reason	Mechanism	Influence	Solution
**Method**		Pre-fractal	Scaling range	Analytical conclusions	Select the most suitable method
**Study area**	Size of study area	Pre-multifractals	Multi-scaling pattern and range	Analytical objects	Define a comparable scope
	Place of study area	Pre-multifractals	Multi-scaling process and range	Analytical objects	Define a comparable location

**Table 8:** Diversity of methods for estimating model parameters or finding solutions to problems.

Type	Model	Methodology	
		Category	Approach
Characteristic Scale	Regression analysis	Algorithm	Least squares, Maximum likelihood, Major axis, Reduced major axis, …
	Factor	Extraction	Principal components, Unweighted least squares, Generalized least squares, Maximum likelihood, Principal axis factoring, Alpha factoring, Image factoring, …
		Rotation	None, Quartimax, Varimax, Equamax, Promax, Direct oblimin, …
		Analytical base	Correlation matrix, covariance matrix
	Hierarchical cluster	Cluster	Between-groups linkage, Within-groups linkage, Nearest neighbor, Furthest neighbor, Centroid clustering, Median clustering, Ward’s method, …
		Measure	Euclidean distance, squared Euclidean distance, cosine, Pearson correlation, Chebychev distance, Block distance, Mahalanobis distance, Minkowski distance, varied customized distance,
		Value transform	None, standardization (Z scores), range standardization (range –1 to 1), range normalization (range 0 to 1), maximum magnitude of 1, mean of 1, standard deviation of 1, …
	Auto-regression	Algorithm	Exact maximum-likelihood, Cochrane–Orcutt, Prais–Winsten, Least squares, …
	Spatial autocorrelation	Measurement	Moran’s *I*, Geary’s *C*, Getis’ *G*, Ripley’s *K*, …
		Calculation	Conventional formula, Three-step calculation, Matrix scaling, Standard deviation, Least square, …
		Contiguity matrix	Power function, exponential function, step function, …
Scaling	Fractals	Algorithm	Least squares, Maximum likelihood, Major axis, Reduced major axis, …
		Measurement	Box-counting, sandbox, radius scaling, radius of gyration, walking divider, geometric measure relation, spectral analysis, distribution function, …
